# Descriptive study of the haematological management of adult patients with severe respiratory failure receiving venovenous extracorporeal membrane oxygenation

**DOI:** 10.1186/cc14383

**Published:** 2015-03-16

**Authors:** O Tavabie, R Pocock, N Barrett, A Retter

**Affiliations:** 1 Guy's and St. Thomas NHS Foundation Trust, London, UK

## Introduction

Venovenous extracorporeal membrane oxygenation (VV-ECMO) is a novel therapy for severe respiratory failure (SRF). Its Introduction has reduced mortality; however, patients require substantial blood product support and between 10 and 20% of cases develop a life-threatening haemorrhage.

## Methods

We contacted 336 practitioners at 135 centres, examining their haematological management.

## Results

In total 25% of practitioners contacted responded; 85% were attending physicians, predominantly based in North America and Europe, 41 and 32% respectively. Ninety-six per cent of units used a polymethylpentene membrane oxygenator and all used a centrifugal pump. Thirty-four per cent of responders managed <10 cases a year and 60% worked in units handling <20 annually, 6% saw >50 patients. One centre did not use unfractionated heparin. Monitoring of anticoagulation varied; 52% used the APTT, 43% the ACT and 5% the APTTr. Sixty per cent did not routinely measure antithrombin. Scenario 1 was based on a patient with H1N1. Practitioners targeted a haemoglobin (Hb) of 80 to 100 g/l; however, 20% targeted a Hb outside this range; 38% favouring a transfusion threshold of <80 g/l when the patient was improving compared with 32% when the patient had just started on ECMO. Seventeen per cent of practitioners transfused platelets when the count was <30 × 10^9^/l whilst 21% maintained the platelet count >100 × 10^9^/l. Scenario 2 described a patient with SRF secondary to a hospital-acquired pneumonia. The patient developed a haemothorax, with persistent blood loss of 200 ml/hour. Practitioners targeted a higher haemoglobin concentration of 100 g/l and targeted a higher platelet count of >100 × 10^9^/l when compared with the patient in scenario 1, neither of these differences was statistically significant. Seventy-one per cent stated they would manage the patient off anticoagulation. There was no agreement as to the length of time off anticoagulation; 26% restarted anticoagulation in <12 hours, compared with 22% who advised no anticoagulation for >5 days. Scenario 3 examined the management of an incidental intracranial haemorrhage. There was a lack of consensus regarding the duration off anticoagulation; 14% of responders held anticoagulation for less than 12 hours whilst 37% held anticoagulation for >5 days and tranexamic was considered useful by 25%.

## Conclusion

There was wide variation in the use of blood products and the intensity of anticoagulation. This is not surprising given the current lack of evidence. Further work is required to provide a standardised approach.

